# Epigenetics and metabolism at the crossroads of stress-induced plasticity, stemness and therapeutic resistance in cancer

**DOI:** 10.7150/thno.42523

**Published:** 2020-05-15

**Authors:** Dinoop Ravindran Menon, Heinz Hammerlindl, Joachim Torrano, Helmut Schaider, Mayumi Fujita

**Affiliations:** 1 Department of Dermatology, University of Colorado School of Medicine, Aurora, CO, USA; 2 The University of Queensland Diamantina Institute, The University of Queensland, Brisbane, OLD, Australia; 3 Eastern Colorado VA Health Care System, Aurora CO, USA; 4 Department of Immunology and Microbiology, University of Colorado School of Medicine, Aurora, CO, USA

**Keywords:** Epigenetics, metabolism, stress-induced plasticity, stemness

## Abstract

Despite the recent advances in the treatment of cancers, acquired drug resistance remains a major challenge in cancer management. While earlier studies suggest Darwinian factors driving acquired drug resistance, recent studies point to a more dynamic process involving phenotypic plasticity and tumor heterogeneity in the evolution of acquired drug resistance. Chronic stress after drug treatment induces intrinsic cellular reprogramming and cancer stemness through a slow-cycling persister state, which subsequently drives cancer progression. Both epigenetic and metabolic mechanisms play an important role in this dynamic process. In this review, we discuss how epigenetic and metabolic reprogramming leads to stress-induced phenotypic plasticity and acquired drug resistance, and how the two reprogramming mechanisms crosstalk with each other.

## Introduction

Acquired drug resistance is one of the major causes of mortality in cancer. This phenomenon is wide spread for distinct kind of treatment strategies which include standard chemotherapy, targeted therapy and immune therapy. Multiple mechanisms have been implicated in the development of drug resistance, which include reactivation of targeted pathways, activation of parallel pathways, drug efflux, and immune evasive mechanisms 1 . While earlier studies have suggested a Darwinian model of evolution with a rigid non-reversible phenotype caused by genetic alterations and natural selection, others have shown a reversible resistance phenotypic state and the involvement of non-Darwinian factors 2-6. The latter model points to a more dynamic process that involves tumor microenvironment, cellular heterogeneity and phenotypic plasticity, all of which play an important role in the evolution of acquired drug resistance 7, 8. These non-Darwinian factors may be distinct, but are often interconnected with phenotypic plasticity. This process of phenotypic plasticity has many similarities to genetic accommodation or “organic selection”, a term coined by Baldwin 9, except that some of these adaptations are heritable in cancer cells, which could be explained by the process of epigenetic imprinting. Phenotypic plasticity provides cancer cells an initial survival advantage that allows them to accumulate genetic changes or imprint epigenetic memory, leading to a resistant phenotype. The initial underlying mechanisms aiding this process could be our own pre-existing cellular programs that allow our body to cope with a stressful or unfavorable microenvironment, which is adapted by cancer cells to acclimate to natural or drug-induced challenges 10.

This process of cellular plasticity is not only the privilege of cancer cells but also shared by non-cancerous cells. While differentiation and heterogeneity of human stem cells were initially thought to be unidirectional with embryonic stem cells differentiating into adult stem cells and differentiated tissues, growing evidence suggests that each of these phenotypes is semi-stable and interchangeable 11. Accordingly, differentiated cells from normal tissue can acquire distinct phenotypes, as shown by trans-differentiation experiments where distinct cell types are inter-convertible without reprogramming into an adult stem cell or embryonic stem cell phenotype 12. Similarly, cancer cells could be in diverse semi-stable epigenetic states with more intrinsic plasticity, allowing them to switch between phenotypes, depending on fitness landscape or mutation-selection balance 13-17.

Whether it is a cancer or non-cancerous cell, the primary factor that defines a metastable (stable states of a dynamic system) or differentiated state is the pattern of gene expression. Hence, epigenetic memory plays an important role in stabilizing each state or transitioning from one state to another. Metabolic factors also trigger and support the transition. Both processes of epigenetic memory and metabolic changes are mutually regulated in nature rather than unidirectionally controlled 18. These two mechanisms are also important in developing and maintaining cancer subpopulation with stem-like characters, such as high tumor growth potential, drug resistance and tumor heterogeneity 19, 20. In this review, we will discuss how epigenetic and metabolic factors drive phenotypic plasticity in cancer, and highlight their importance in cancer stemness, disease progression and drug resistance.

## Cancer Stemness, Cellular Stress and Senescence

Stem cells are classically defined by two basic principles; the ability to self-propagate and the potency to differentiate into multiple cell types 21. Similarly, cancer stem cells (CSCs) are historically defined by two fundamental stem cell properties; self-propagation and restoration of the original tumor with phenotypic heterogeneity 22, 23. Another feature of CSCs is their resilience to unfavorable microenvironments and drugs, which allows them to survive in disadvantageous conditions and re-establish the tumor 24. The initial CSC model of a unidirectional hierarchical system was established from the studies of hematological cancers where rare cell populations propagated after serial transplantations 25. Accordingly, a variety of markers were identified in each cancer type that met the defining criteria for CSCs 23. However, recent studies have provided evidence for a dynamic, non-hierarchical cancer stemness model in multiple cancer types including glioblastoma, breast cancer, pancreatic cancer and melanoma by accommodating the concept of phenotypic plasticity 26-31. Therefore, subpopulations with rapid tumor-initiating potency can rise from stochastic switching of cancer cell populations 31. While it is clear that subpopulations of cells have growth advantage or drug resistance capability when compared to the bulk of tumor cells, it is likely that these populations are in flux through constantly switching phenotypes.

These studies also raise another interesting question about cancer stemness. Can a particular phenotype qualify as stemness or can the process of plasticity itself better explain the stemness? While some of these phenotypes have a growth or survival advantage in certain conditions, this advantage could be rather contextual, entailing cells to switch between these states to grow and thrive. Often, cells in a slow-cycling or semi-quiescent phenotype are resistant to a wide variety of treatments and described as cancer persister cells 32. They are also reported to have an advantage in tumor growth and tumor-initiating potential 33. This observation is rather counterintuitive as it is difficult to directly explain how a slow-cycling population can lead to higher tumorigenicity. A possible explanation would be that these cells enter a state that is more tolerant to a wide variety of stressful conditions, which allow their initial successful engraftment and subsequent swift switch into a proliferative state once the environment becomes favorable. On the other hand, cells already in a proliferative state may need to switch into a slow-cycling state to allow their engraftment and then switch back to a proliferative state for tumor growth. This explanation points to a general mechanism whereby a slow-cycling state, described as a pseudo-senescent or quiescent phenotype, can be associated with cancer stemness.

Cellular senescence is broadly classified into three types: replicative senescence, embryonic senescence and stress-induced premature senescence (SIPS) 34. Replicative senescence is characterized by shortening of telomeres due to cellular replication, a closely regulated process to ensure tissue homeostasis 35. Embryonic senescence, on the other hand, plays a central role in early embryonic development. While embryonic senescence was thought to be terminal, recent studies suggest it is temporal in nature and cells exit out of senescence at later stages 36. SIPS, as the name suggests, is a stress-induced premature senescence phenotype that does not affect telomere function 37. SIPS is induced by cellular stress from extrinsic and intrinsic factors such as oncogenic stress, chemotherapeutic agents, ionizing radiation and nutrient starvation 38-40. Hence, SIPS have characteristics that resemble the senescent-like phenotype observed in cancer cells that arise due to oncogenic stress or treatment with therapeutic agents.

The underlying process relating these seemingly paradoxical phenotypes of senescence and cancer stemness had remained elusive. However, a recent study illustrated that the process of therapy-induced senescence promoted cancer stemness by reprogramming cells and enhancing canonical WNT signaling 41. This phenotypic shift from senescence into a stem-like state could be a universal phenomenon not exclusive to cancer cells, since even 'Yamanaka factors' 42 induces an initial transition of primary cells into senescence that is required for efficient reprogramming 43, 44. Because cancer cells are heterogeneous in their efficacy to switch between slow-cycling persisters and proliferative states, the cells that are highly dynamic and switch effortlessly between these states could have growth and survival advantages over others.

**Figure 1:**
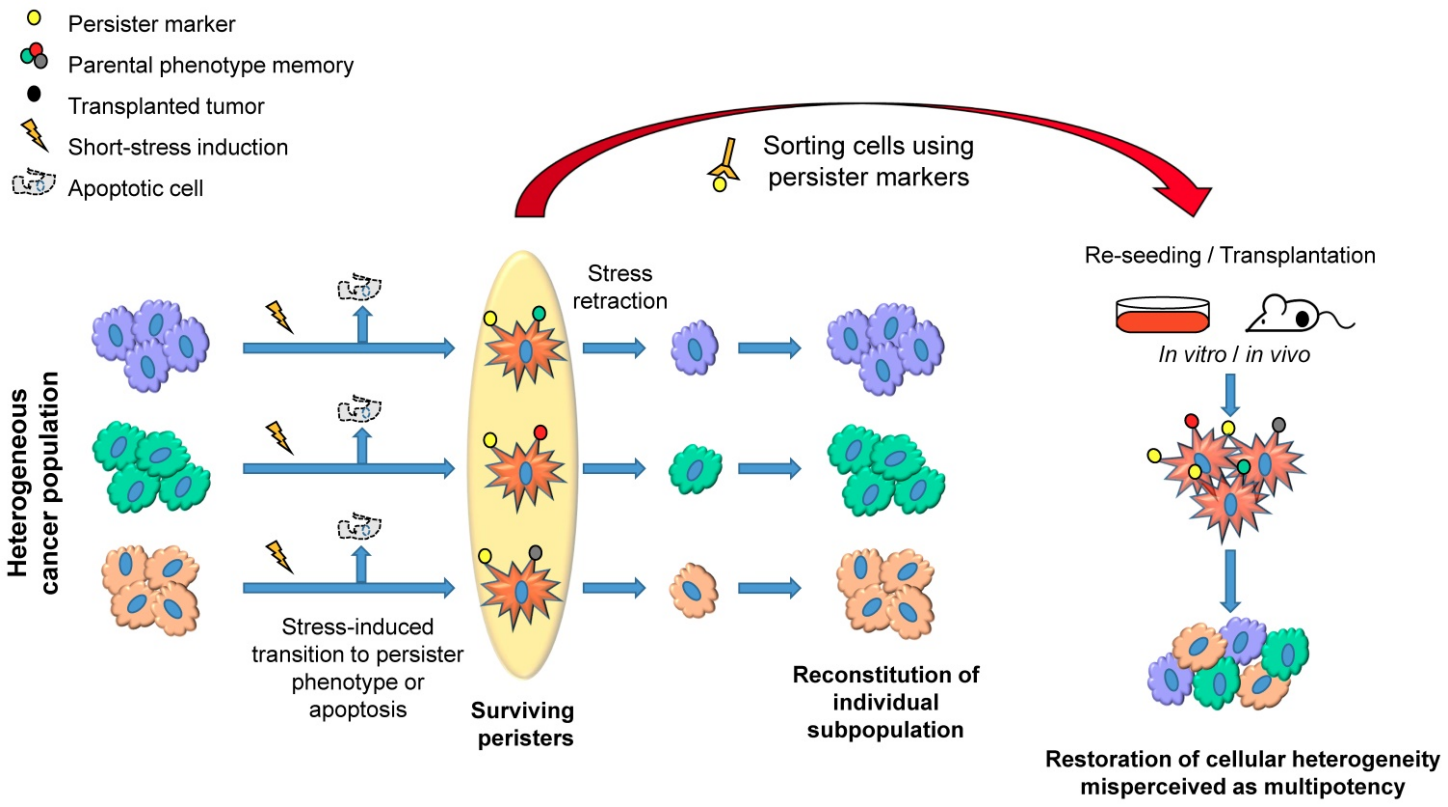
A schematic representation describing how distinct subpopulations of cancer cells transiently shift into a persister state (star-shaped red cells) under stress and maintain tumor heterogeneity. The short-term stress exposure causes a shift of multiple subpopulations into persister phenotype both in vitro and in vivo. The efficiency of transition could vary depending on the stress-inducing conditions and sensitivity of subpopulations to the stress. When the factor inducing stress is removed, the surviving persisters would reverse back to their original phenotypes and proliferate to reconstitute their subpopulations (left). When persister populations are sorted using persister markers and reseeded in vitro or implanted in vivo, each of the populations reverse back to the original phenotype, resulting in the restoration of phenotypic heterogeneity, which could be misperceived as pluripotency or multipotency of cancer cells, because it looks as if the persister cells differentiate into multiple phenotypes (right).

In line with these observations, we have shown that acute stress-induced phenotypic switching of distinct melanoma subpopulations into a slow-cycling state 45. This switching led to the upregulation of multiple melanoma stem cell markers including nerve growth factor receptor (NGFR) 46, ATP-binding cassette sub-family B member 5 (ABCB5) 47 and aldehyde dehydrogenase (ALDH) activity 48. However, this process did not involve direct reprogramming, and the slow-cycling cells reverted back to their respective phenotype once the stress was removed, suggesting that the cancer persister cells serve as a reservoir for many distinct subpopulations, thereby maintaining tumor heterogeneity (Figure 1, left). We have also shown that this switching into persister cells provided stem-like characteristics such as high tumorigenic potential and drug resistance 45. Therefore, the markers of a slow-cycling state fulfill the definitions of the hierarchical CSC model except lineage hierarchy (Figure 1, right). This concept is supported by many studies, which show that unfavorable conditions, such as hypoxia, drug treatment and nutrient starvation, induce phenotypic plasticity that leads to the induction of a stem-like state in cancer cells 45, 49-54. The transition into a slow-cycling state is also closely associated with an epithelial-mesenchymal transition (EMT) that leads to drug resistance and metastasis 55-57. Drug holiday studies have shown that the slow-cycling populations swiftly switch back into a proliferative state in the absence of drug and display phenotypes corresponding to cancer stemness 2, 3, 45, 55, 58, 59. Accordingly, CSCs are often reported to be in a slow-cycling state, providing them with drug resistance and tumor growth advantage 60.

## Markers Identifying Cancer Persisters

The slow-cycling, drug-resistant persister population has been detected in multiple cancer types and often shares previously described markers of cancer stem cells. However, it is unclear whether these markers are related with the persister phenotype. Below, we will discuss some of the markers that have been shown to identify cancer persisters. Members of the jumonji AT-rich interactive domain-1 (JARID1) family, particularly lysine-specific demethylase 5A (KDM5A) and lysine-specific demethylase 5B (KDM5B) 2, 3, 45, 58, 59, 61, demethylate histone 3 lysine 4 trimethylation (H3K4me3). KDM5A and KDM5B expression correlates with the expression of stem cell markers in various cancer types 2, 45, 62. Many of these studies explain the rise of a slow-cycling population as an outcome of random stochastic switching within the population 2, 3. However, we have shown that chronic stress and drug treatments induce the upregulation of KDM5A and KDM5B in multiple cancer types 45, 63. KDM5 gene is shown to modulate oxidative stress response in a Drosophila model, therefore its upregulation would illustrate an evolutionarily conserved mechanism for coping with stress 64. Indeed, KDM5A plays an important role in hypoxia-induced chromatin reprogramming 65 while hypoxia-inducible factor HIF1α is a direct transcriptional regulator of KDM5B 66. In line with the correlation of KDM5 with a slow-cycling state, H3K4me3 demethylation by KDM5A and KDM5B is associated with cellular senescence 67, indicating a conserved mechanism for KDM5 to cope with stressful environments. However, KDM5B was also shown to prevent terminal differentiation of embryonic bodies 68. Therefore, KDM5 could be a double-edged sword, acting initially as tumor suppressor and senescence inducer but later driving cancer progression.

Another key marker is NGFR, which was shown to be associated with stemness in melanoma 46, 69, breast cancer 70, colon cancer 71 and squamous cell carcinoma 72, 73. NGFR expression is transient 45, 74, similar to that of KDM5A and KDM5B, and represents a slow-cycling phenotype in multiple cancer types including melanoma 45, breast cancer 75, lung cancer 63 and squamous cell carcinoma 73. The induction of NGFR by interferon gamma (IFN-γ) in cancer cells 76 suggests a generic stress-induced phenotype. We have shown that NGFR expression is indeed induced by multiple stress factors such as drug exposure, hypoxia and glucose starvation in multiple cancer types 45, 63 and correlates with KDM5A/B expression. In many cancers, NGFR expression correlates with ALDH activity 45, 70, 77, which is upregulated in cancer persisters 78, 79. However, since ALDH activity is regulated by a family of ALDH genes 80, its applicability to consistently identify persister population needs further testing. NGFR inhibits p53 activity 81 and protects cells from reactive oxygen species 82, which would account for the drug resistant mechanism of NGFR. NGFR expression is associated with resistance to MAPK inhibitors (MAPKi) in melanoma 45, 83 and chemotherapy in multiple cancer types 81, 84. NGFR expression is also associated with downregulation of melanoma antigens, suppression of cytotoxic T cell responses 76 and resistance to adoptive T cell transfer therapy in melanoma patients 85 and mouse models 86. Taken together, these studies indicate that a stress-induced shift to a slow-cycling state of cells expressing KDM5 and NGFR serves as a crucial step for cancer progression and therapy resistance. We will further discuss how the stress-induced expression of these factors contribute to disease progression through epigenetic reprogramming in the section below.

## Role of stress-induced epigenetic reprogramming in cellular plasticity and drug resistance

Epigenetics modifies genes via several mechanisms including DNA methylation and histone modifications. Epigenetic modulation plays a central role in phenotypic plasticity, and determines cell fate in many processes such as senescence induction and maintenance, important steps for slow-cycling cancer cells. The stress-induced, slow-cycling subpopulation of cancer cells carries a resemblance to a SIPS phenotype of non-cancerous cells. SIPS is regulated through temporal histone modifications rather than DNA methylation processes 40, 87, whereas replicative senescence is controlled by heritable DNA methylation changes such as global hypomethylation 88 of DNA and focal DNA hypermethylation 89. The modifications in SIPS include a loss of transcriptional activator mark, H3K4me3 67, 90, and a gain of transcriptional repressor mark, histone 3 lysine 9 trimethylation (H3K9me3) 91, 92, both of which result in repressive chromatin formation and gene silencing. H3K9me3 plays an important role in the formation of senescence-associated heterochromatin foci in replicative senescence, embryonic senescence and SIPS 92-94, suggesting their generic role in multiple senescent phenotypes. Accordingly, a shift to a slow-cycling stem-like state in cancer cells is accompanied by a loss of 2, 45, 58, 63. The H3K4me3 demethylation is carried out by KDM5A and KDM5B 2, 45, 58, whereas the modification of H3K9me3 is achieved by two histone methyltransferases, SET domain bifurcated histone lysine methyltransferase 1 (SETDB1) and SET domain bifurcated histone lysine methyltransferase 2 (SETDB2) 63, 90. SETDB1 is involved in cellular senescence through formation of heterochromatin structures, which then leads to alternative lengthening of telomeres and cancer cell immortality 95. In line with this, SETDB1 has been shown to contribute to melanoma progression and therapy resistance 96. On the other hand, the role and function of SETDB2 in the process of senescence and slow-cycling cancer cells remain largely unknown. In addition, despite the above-mentioned important roles for histone modifications in a slow-cycling stem-like state in cancer cells, no correlation exists between DNA methylation patterns and gene expression in these slow-cycling cancer cells nor are the DNA methylation patterns associated with this shift 63. This is not surprising, as changes in DNA methylation patterns are considered to be more stable and heritable than histone modifications, which would explain the transient and reversible phenotype of a slow-cycling state. Subsequently, the H3K4me3 and H3K9me3 epigenetic patterns are reversible when the stress exerted on the cells is released. Furthermore, while epigenetic phenotypes of low H3K4me3 and high H3K9me3 are commonly observed in various cancer types, the gene expression profiles altered by the shared epigenetic patterns are cancer type-specific rather than universal 63. This is expected, as different cancer types need to upregulate or downregulate a unique set of genes to switch into a slow-cycling cancer persister state.

When cancer cells are treated with drugs continuously, the slow-cycling phase leads to an acquired drug-resistant state that is phenotypically stable compared to the transient, slow-cycling state 4, 45, 83. This observation suggests that the slow-cycling state is important for two distinct functions: The first being its role in ensuring survival of the cell population after acute stress and the second being its capacity to develop a stable drug resistant phenotype. This could happen either through the gain of genetic changes or epigenetic cellular reprogramming in the slow-cycling state. Recent studies have demonstrated the involvement of the latter process in the development of acquired drug resistance that is phenotypically stable. For example, when melanoma cells were treated with MAPKi for a long period, they underwent global epigenetic reprogramming and upregulated the expression of drug resistant genes such as APC down-regulated 1 (*APCDD1*), epidermal growth factor receptor (*EGFR*), platelet-derived growth factor receptor beta (*PDGFRB*) and neuregulin 1 (*NRG1*). This process was gradual and dependent on the continuous exposure to the drug, resulting in a shift from a transient transcriptional state to a stable resistant state 83. Accordingly, escape of cancer cells from drug-induced senescence was associated with a gain of specific H3K4me3 peaks that lead to CSC gene activation 41, which could aid global epigenetic reprogramming. The involvement of transcriptional reprogramming in the escape of cancer cells from a slow-cycling state to a stable resistant state was also reported in other melanoma studies 97, 98, one of which shows an association of this reprogramming with histone 3 lysine 27 (H3K27) acetylation and a mesenchymal phenotype 97. H3K27 acetylation is a transcriptional activator and negatively correlates with DNA-methylated CpG islands that correspond to gene inactivation 99. These studies suggest that even though transient subpopulations of preexisting cells have an advantage against inhibitors during the initial selection process, they further undergo a time-dependent epigenetic reprogramming to acquire a stable resistant state. Interestingly, while the stable resistant phenotype showed substantial changes in the global access to chromatin, the epigenetic changes in transient resistant cells were only marginal in comparison to untreated cells, suggesting its gradual shift over the course of treatment 83. These observations indicate that multiple interconnected epigenetic mechanisms could be involved in global epigenetic remodeling. They also suggest that DNA methylation could have a decisive role in determining stable resistant phenotypes, even though its involvement is limited in a transient resistance mechanism. Non-genetic progressive transformations leading to drug resistance were also reported in lung cancer patients who underwent EGFR inhibitor therapy 100, 101. The underlying mechanism that leads to epigenetic reprogramming is not known. Furthermore, while the early transient phenotype is well documented in certain cancer types, whether this early phenotype directly contributes to acquired stable resistance is still a topic under investigation 102.

When cancer cells are treated with drugs continuously until they acquire drug resistance, stopping the drugs thereafter can lead to sudden hyperactivation of pathways and fitness deficit in the resistant populations 4, 5, 103, 104. However, this growth disadvantage of resistant cancer cells could be overcome by epigenetic or transcriptional remodeling, resulting in the development of a stable drug-resistant phenotype and tumor progression. For example, dual specificity phosphatase 5 (DUSP5) is required for recalibrating MAPK activation in cancer cells that have accrued B-Raf proto-oncogene, serine/threonine kinase (*BRAF*) mutations 105. Similarly, SHOC2 scaffold protein is needed for *NRAS ^Q61K^*-induced resistance to BRAF inhibitors 106. These observations suggest that epigenetic reprogramming is utilized by resistant cancer cells to negate the fitness deficit induced by genetic changes, which, cells gradually evolve to defeat through factors that help them to recalibrate the activation of signaling pathways. Therefore, in order to control drug-resistant cancer cells that have acquired resistant genetic changes, the strategy to administer drugs intermittently would be effective, because 'drug holidays' would result in the loss of fitness in the resistant cells and subsequent outgrowth of drug-sensitive parental cells 4. These mechanisms attribute to the success of 'drug holidays' in MAPKi resistance in melanoma and EGFRi resistance in lung cancer patients 4, 5, 103, 104, 107. Furthermore, 'drug holidays' could prevent transiently resistant cancer cells from acquiring a stable resistant phenotype, as stable epigenetic/transcriptional reprogramming is usually achieved through continuous drug exposure 83, 108.

In addition to the resistance to signaling inhibitors, stress-induced phenotypic plasticity may be involved in resistance to PD-1 checkpoint inhibition. Melanoma tumors that acquire BRAF/MEK inhibitor resistance are shown to display a mesenchymal phenotype and an immunologically-cold, M2 macrophage signature with decreased infiltration of cytotoxic CD8 T cells compared to the pre-treatment tumor 109, 110. Accordingly, BRAF/MEK inhibitor resistance signatures correspond with an innate resistance to anti-PD-1 therapy signature, and share many factors that induce immune escape, including AXL receptor tyrosine kinase (AXL) 111, vascular endothelial growth factor (VEGF) 112, IL8 113 and C-C motif chemokine ligand 2 (CCL2) 114, 115. These observations could explain why patients who progress under BRAF/MEK inhibitors respond poorly to immune checkpoint inhibition 116-118. In line with these reports, the expression of KDM5B in cancer cells, corresponding to the slow-cycling phenotype, leads to the suppression of anti-tumor immune responses by inducing EMT, suppressing interferon response signaling pathways and downregulating cancer testis antigen expression, all of which are important for immune surveillance 119, 120.

**Figure 2:**
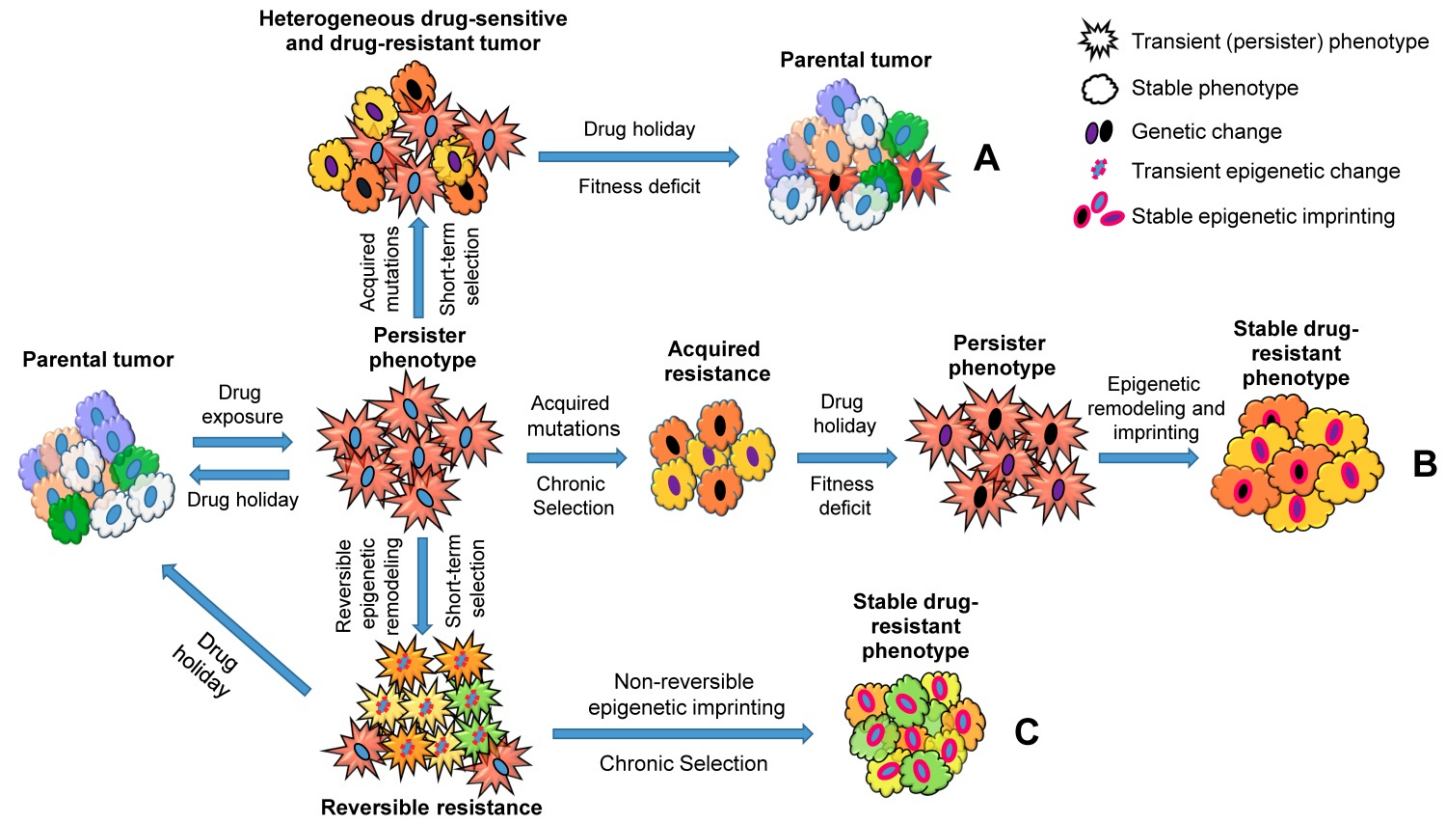
A schematic representation describing how cancer persister cells contribute to therapy resistance through genetic and epigenetic changes. Parental cell populations under drug treatment acquire a dormant persister phenotype (star-shaped red cells). The transition is transient in nature and dependent on continuous drug exposure. The persister cells can acquire mutations that produce resistant subpopulations (A and B: Mutant subpopulations are shown using the changes in nuclear and cytoplasmic colors, representing genetic and phenotypic changes, respectively). Under a short-term drug selection followed by a drug holiday, the mutant drug-resistant subpopulations shift their phenotypes into dormant persisters due to the lack of fitness in the absence of drug, and non-mutant cells in turn switch back to parental subpopulations (A). On the other hand, under a chronic drug selection, the mutant subpopulations outgrow the non-mutant populations; however, under a drug holiday, the lack of fitness in the mutant populations leads to a persister phenotype that could be overcome by epigenetic reprogramming (Epigenetically reprogrammed subpopulations are shown using the change in nuclear membrane colors to pink). The final phenotype observed in this process is stable and does not respond to drug treatments or drug holiday (B). In contrast to the genetic changes observed in A and B, the dormant persister cells could also undergo epigenetic remodeling to acquire a semi-proliferative transient resistant state (multi-colored star-shaped population with pink dotted nuclear membrane). When these cells are treated with drugs continuously, they can further undergo epigenetic imprinting to transition into a resistant phenotype (C, right. Epigenetically reprogrammed subpopulations are shown using the change in their nuclear membrane color to pink). Similar to the resistant cells caused by genetic changes (B), the final phenotype observed in the process (C) is stable and does not respond to drug treatments or drug holiday. However, a drug holiday before establishing this stable phenotypes could prevent semi-proliferative transiently resistant cells from acquiring stable resistance (C, left).

Taken together, these studies suggest a central role of stress-induced phenotypic plasticity that contributes to acquired drug resistance (Figure 2). This could be achieved through slow-cycling persister cells either gaining mutations (Figure 2; A and B) or undergoing epigenetic reprogramming (Figure 2; C). When cancer cells receive a short-term drug treatment, 'drug holidays' induce persister cells to differentiate back to the parental tumor phenotype and outgrow the cancer cells that acquired drug-resistant genetic changes, because the latter cells lose fitness and will acquire a persister phenotype in the absence of the drug (Figure 2; A). However, when cancer cells receive a long-term treatment, the fitness deficit induced by 'drug holidays' would be overcome by epigenetic modelling, and cancer cells regain cellular homeostasis (Figure 2; B). In addition, long-term exposure of drugs would induce phenotypic plasticity and stable drug resistance in cancer cells without genetic changes (Figure 2; C). Overall, a ubiquitous stress-induced epigenetic plasticity plays a critical role in the evolution of cancer drug resistance and disease progression. The mechanisms driving this important shift still remains to be elucidated.

## Metabolic remodeling during phenotypic plasticity and therapy resistance

Metabolic adaption is a hallmark of cancer development 121 and significantly contributes to stem cell reprogramming and epigenetic regulation 122-124. The importance of metabolism for cellular plasticity is evident by the vastly different metabolic states of embryonic stem cells (ESCs) compared to most differentiated cells. ESCs rely on high rates of glycolysis and have poorly developed, highly fragmented mitochondria that mature during differentiation, coinciding with a switch from glycolysis to oxidative phosphorylation 125. The critical role of metabolism in ESCs is also evident in stemness transcription factor-induced cellular reprogramming 126. In fact, the generation of induced pluripotent stem cell (iPSC) is accompanied by mitochondrial remodeling, resulting in immature spherical and cristae-poor structures and a glycolysis-dependent metabolic phenotype 126 that is, at least in part, regulated by *de novo* fatty acid synthesis to facilitate mitochondria fission 127.

Despite the morphologic evidence that suggests underdeveloped mitochondria in stem cells, human pluripotent stem cells (hPSC) mitochondria are capable of respiration at maximal capacity, with the mitochondria uncoupling protein UCP2 preventing glucose-derived pyruvate oxidation 128. Moreover, glutamine oxidation is crucial for the maintenance of TCA cycle intermediates and ultimately hPSC survival 129. Overall, hPSC show a substantial plasticity in their metabolic program whereas their energy requirements mainly depend on glycolysis, with mitochondria metabolism playing crucial roles for survival and cell fate decisions 124, 130. Similarly, cells that transition to a senescent phenotype undergo extensive metabolic remodeling that can lead to increased glycolysis and/or oxidative phosphorylation 131.

One of the key events in oncogene-induced senescence is a specific shift of pyruvate utilization toward the TCA cycle 132. *BRAF^V600E^*-induced senescence in human fibroblasts activates pyruvate dehydrogenase (PDH) through suppression of pyruvate dehydrogenase kinase 1 (PDK1) and induction of pyruvate dehydrogenase phosphatase 2 (PDP2), resulting in cell cycle arrest and senescence 132. Overexpression of PDK1 allows p53-depleted *BRAF^V600E^*-expressing melanocytes to develop tumors, whereas PDK1 knockdown in melanoma reduces tumor initiation, progression and maintenance 132, supporting a tumor-promoting role of PDK1. The requirement of a PDK1-mediated switch, in and out of a senescence-like state, to promote tumor initiation and maintenance is reminiscent of the KDM5B^high^ slow-cycling melanoma subpopulation that drives tumor initiation, propagation and drug resistance 3, 58. KDM5B^high^ cells are also dependent on mitochondria metabolism with increased dependence on glucose and fatty acids to fuel their oxidative phosphorylation 3, 133. Interestingly, PDK1 knockdown sensitizes *BRAF*-mutant melanoma to BRAF inhibitor treatment 132, suggesting the role of senescence PDK1-mediated drug resistance. It would be interesting to test how PDK1 affects markers of cancer persisters, such as KDM5B and NGFR, and whether the knockdown cells are still capable of stochastic phenotypic switching. What we do know is that the emergence of drug tolerant melanoma cells is accompanied by a BRAF inhibitor-induced suppression of glycolysis 134 as well as dependence on mitochondrial biogenesis and oxidative phosphorylation 3, 135. A similar reliance is found in stem-like drug resistant subpopulations of chronic myeloid leukemia 136, acute myeloid leukemia 137, breast cancer 138 and glioblastoma 139, suggesting that a metabolic switch toward a mitochondria-dependent phenotype is common for therapy-resistant stem-like cancer cells. A consequence of increased oxidative phosphorylation is, among others, tissue hypoxia due to increased oxygen consumption, which is associated with T-cell exhaustion and therefore, decreased anti-PD-1 response in melanoma mouse models 140. This effect can be overcome by oxidative phosphorylation inhibitors such as metformin 141 that target metabolic adaptions to boost immunogenicity. A similar shift toward oxidative phosphorylation is observed in melanoma brain metastases, and linked to immunosuppression and brain metastasis incidence 142. It is worth noting that glycolysis-related gene expression patterns and tumor glycolytic activity have been negatively correlated with T-cell infiltration, leading to adoptive T-cell therapy failure in melanoma and lung cancer 143. This is in part mediated by tumor-derived lactate, a byproduct of anaerobic glycolysis in the tumor microenvironment, that results in the suppression of natural killer cell cytolytic function 144 and polarization of tumor-promoting macrophages 145, suggesting that a balance between oxidative and glycolytic metabolism is important to evoke an effective anti-tumor immune response.

An intriguing feature of slow-cycling CSCs in glioblastoma and ovarian cancer is a specific increase in unsaturated lipid metabolites 139, 146 that was later found to be a therapeutic vulnerability 146. This resembles the recently identified increase of unsaturated fatty acids in the persister cells of various cancer types after treatment with anti-cancer drugs 55, 147, which leads to a reliance on enzymes involved in the detoxification of lipid peroxides, mainly glutathione peroxidase 4 (GPX4) to prevent ferroptosis 55, 56, 147, 148. This vulnerability to iron-dependent oxidative stress is also found in response to immune therapy 98. Mechanistically, ferroptosis requires high levels of polyunsaturated fatty acids (PUFA) containing phospholipids in the cell membrane, which are prone to oxygenation 149, suggesting that the observed increase in unsaturated fatty acids and the resulting GPX4 dependence is caused by increased unsaturation of cancer persister cell membranes. In clear cell carcinoma, a highly aggressive form of kidney cancer, HIF-2α was identified as a driver of PUFA generation, a factor that has been described to stabilize β-catenin that mediates lipid desaturation 148, 150-153. Interestingly, WNT-beta-catenin signaling and a permissive H3K4me3 epigenetic landscape at WNT and stemness-related genes are key mechanisms involved in senescence-associated cancer cell reprogramming 41. Accordingly, fatty acid unsaturation has been described to be increased in replicative senescence 154 and oncogene-induced senescence 155, which is also reflected by increased lipid peroxidation in therapy-induced senescent cells 156, suggesting that a senescence-like metabolic remodeling process is involved in cancer persistence.

One functional consequence of the ferroptosis-sensitive state is immunoevasion, possibly through the release of immunosuppressive eicosanoids such as prostaglandin E2 157. Furthermore, the presence of oxidatively-truncated lipids has been shown to interfere with major histocompatibility complex (MHC)-mediated antigen cross-presentation by dendritic cells in cancer 158, and alterations of the membrane lipid composition, specifically increased PUFA content, have been shown to negatively affect antigen presentation and modulate the inflammatory eicosanoid metabolites 159, suggesting decreased immunogenicity of cancer persister cells. Besides changes in lipid metabolism, alterations in amino acid metabolism, specifically tryptophan, have significant immunosuppressive consequences. Tryptophan is an essential amino acid that shows increased uptake in tumor tissue 160 . Recently, the MYC-dependent upregulation of the tryptophan transporters SLC7A5 and SLC1A5 and the tryptophan catabolizing enzyme arylformamidase (AFMID), involved in the conversion of tryptophan into kynurenine, has been identified as a mechanism underlying increased tryptophan uptake in colon cancer cells 161. Within tumor cells, indoleamine-2,3-dioxygenase (IDO) and tryptophan-2,3-dioxygenase (TDO)-mediated tryptophan catabolism produces the active metabolite kynurenine, an agonist of the aryl hydrocarbon receptor (AhR) that exerts immunosuppressive functions 162. The kynurenine-AhR metabolic circuit has been implicated in the dormancy of stem-like tumor repopulating cells (TRC) in response to IFN-γ stimulation 163. These TRCs actively import tryptophan and produce kynurenine, which is released into the tumor microenvironment where it stimulates PD-1 expression in adjacent CD8+ T-cells in a kynurenine-AhR-dependent manner to decrease the immune response 164 and has therefore attracted vast interest in the development of combination therapies including anti-PD-1 therapy 165. This is in line with the previously mentioned transcriptomic signatures of residual MAPKi-treated melanoma that resembles an innate anti-PD-1 resistance (IPRES) signature 109, 110 and the clinical observations that anti-PD-1 therapy is less effective immediately following BRAF inhibition 116. Overall, these studies suggest that stress-induced phenotypic switching to a drug resistant state mediates cross-resistance to PD-1 immune checkpoint inhibitors, which may be mediated, at least in part, by metabolic remodeling.

## Crosstalk between metabolic remodeling and epigenetic regulation

As previously mentioned, a hallmark of cancer persisters is remodeling of their epigenetic landscape 45, 63, 79, a process that is inherently linked to metabolism 166. As described above, the persister phenotype contributing to cancer stemness may require multiple metabolic shifts, one allowing the senescence phenotype and the other allowing the escape from senescence. Hence, dynamic regulation of metabolism, which is linked to epigenetics, could be a key factor driving this plasticity. The shift toward oxidative phosphorylation-dependent metabolism in cancer persister cells can have a strong influence on epigenetics, as multiple TCA cycle intermediates directly affect epigenetic reactions. Among the most important metabolites involved in epigenetic regulation is alpha ketoglutarate (α-KG), which is a co-factor of α-KG-dependent dioxygenases of the Jumonji C (JmjC)-family of histone demethylases and ten-eleven translocation (TET)-family of DNA demethylases, both important to maintain stem cell self-renewal 167 and influence early differentiation of human pluripotent stem cells 168. These reactions convert α-KG to succinate, whereas succinate and the structurally similar fumarate, both TCA cycle intermediates, act as competitive inhibitors 169. For example, accumulation of fumarate has been shown to drive EMT through inhibition of TET enzyme-mediated DNA demethylation and subsequent decreased expression of miR-200 170. Accordingly, KDM5 family H3K4 demethylases belonging to JmjC family are known to be dependent on α-KG for their function 171. Considering the central role of KDM5A and KDM5B for the development of cancer persisters 2, 3, it is very likely that the observed shift toward oxidative phosphorylation is, at least in part, necessary to supporting epigenetic remodeling. Besides glucose-derived carbon, glutamine is a key amino acid for cellular energetics that is often used to feed TCA cycle intermediates in cancer cells 172, a phenomenon reported during BRAF inhibitor resistance 173. In contrast, localized glutamine deficiency in solid tumors results in histone hypermethylation of H3K4me3, H3K9me3, histone 3 lysine 27 trimethylation (H3K27me3) and histone 3 lysine 36 trimethylation (H3K36me3) 174. This shift in epigenetic state leads to cancer cell dedifferentiation and BRAF inhibitor resistance because of decreased α-KG availability and consequently, inhibition of JmjC-family histone demethylases. These cells, subjected to α-KG deficiency, show increased expression of cancer persister marker, NGFR. This observation suggests that a delicate balance in α-KG levels is necessary to maintain the histone epigenetic patterns of cells. A shift in this balance seems to trigger a similar response either through histone hyper- or hypo- methylation, which is quite intriguing. For example, this effect could be due to the upregulation of transcriptional repressor marks, H3K9me3, H3K27me3 and H3K36me3, which could override the effect of H3K4me3 upregulation. Further understanding of the role of KDM5 enzymes in the context of histone hypermethylation is needed.

The one carbon donor, S-adenosylmethionine (SAM), regulates histone and DNA methylation, and H3K4me3 is specifically sensitive to fluctuations in SAM concentrations 175. SAM is the main product of the one carbon cycle and its synthesis and regeneration requires amino acid metabolism 176. Changes in methionine availability, the amino acid that is directly converted to SAM, have been shown to influence H3K4me3 peak width and subsequently, gene expression 177. Depletion of methionine is a vulnerability of tumor-initiating lung CSCs as they are characterized by high methionine cycle activity, which, if not maintained, presumably leads to alterations of the epigenetic landscape, blocking tumor initiation and survival of tumor-initiating cells (TICs) 178. This suggests that the availability of SAM may positively regulate the capacity of cancer persisters to exit the slow-cycling state and gain expression of stem cell factors, which could be dependent on H3K4me3 41. Similarly, CSCs specifically require SAM biosynthesis to maintain viability and H3K4me3 marking 179, exemplifying the fundamental connection of metabolism and epigenetic plasticity. Beside methionine, serine has been reported to contribute to the maintenance of the one carbon cycle, supporting *de novo* nucleotide synthesis by fueling the folate cycle 180, also known as serine, glycine, one-carbon pathway 181. This phenomenon has been shown to occur specifically in TICs 182, and has been described during the development of neuroendocrine prostate cancer, the most lethal subtype of castration-resistant prostate cancer. *De novo* serine synthesis that might rely on retrograde flux through glycolysis 183, together with glucose-derived ribose, are major contributors that fuel *de novo* ATP synthesis to drive SAM generation in inflammatory macrophages 184, highlighting the importance of glycolysis to maintain one carbon pools. Beyond that, one carbon metabolism and SAM levels are strongly influenced by mitochondria dysfunctions, which increase serine biosynthesis and affect polyamine and methionine metabolism as a direct result of changes in TCA flux, resulting in DNA hypermethylation and transcriptional changes 185, 186. In general, metabolic flux through the TCA cycle, the pentose phosphate pathway and the serine, glycine, once carbon pathway (SGOCP) are interconnected and it appears that phosphoglycerate dehydrogenase (PHGDH), the enzyme that commits carbon units to *de novo* serine biosynthesis, coordinates this central carbon metabolism 187. An intriguing example for the interplay between these pathways is the identification of the serine-responsive SAM-containing metabolic enzyme complex in yeast 188. This complex consists of the yeast analogs of pyruvate kinase M2, serine metabolic enzymes, SAM synthetases, and an acetyl-CoA synthetase, that interacts with the H3K4 methyltransferase complex SET1 to regulate H3K4me3, amongst other histone modifications 188.

Similar to histone and DNA methylation, histone acetylation and deacetylation are dependent on the availability of metabolic co-factors. Glucose-derived acetyl-CoA is required as a substrate for protein acetylation and is generated in an ATP-citrate lyase (ACL)-dependent manner 189. AKT activation, which is found in response to treatment with anti-cancer drugs 45, 63, facilitates ACL-dependent acetyl-CoA production in low glucose conditions, possibly aiding increased H3K27 acetylation of cis-regulatory elements found in slow-cycling drug tolerant glioblastoma stem cells 190. However, in addition to glucose, acetyl-CoA derived from fatty acids 191 or acetate recycling 192 has been shown to fuel histone acetylation. Detailed analysis of metabolic mechanisms that fuel acetylation reactions during the development of drug resistance is warranted. The reverse reaction, histone deacetylation, is also in partly dependent on the availability of the metabolite nicotinamide adenine dinucleotide (NAD+). Skeletal muscle stem cells undergoing a transition from a quiescent to a proliferative state reprogram their metabolism from oxidative phosphorylation dependent to glycolysis dependent, which results in decreased NAD+ availability and subsequently, increased histone acetylation 193. The importance for NAD+ metabolism for therapy resistance is highlighted by the key roles of NAD+ metabolism for SAM-dependent methylation reactions and glioblastoma stem cell maintenance 194 as well as the dependence of self-renewal and radiation resistance of glioblastoma stem-like cells on nicotinamide phosphoribosyltransferase (NAMPT), the rate-limiting step in NAD+ synthesis 195. Whether or not NAD+ metabolism is important for cancer persistence is currently unclear but the high degree of lipid desaturation discussed previously would be an intriguing avenue that has been shown to contribute to NAD+ recycling 196.

Another interesting caveat is the local synthesis of metabolites to support enzymatic reactions, which has been demonstrated for the nuclear synthesis of fumarate 197 and acetyl-CoA 198 as well as the previously mentioned SAM-containing metabolic enzyme complex 188. A more comprehensive nuclear translocation of TCA cycle enzymes occurs during zygotic genome activation (ZGA) in early embryogenesis, a shift depending on protein O-GlcNAc transferase (OGT) ultimately promoting epigenetic remodeling 199. Interestingly, OGT is known to play an important role in multiple stress responses including oxidative, ER, and genotoxic stresses 200. Chromatin-associated fumarase is phosphorylated by AMPK at Ser75 to maintain di-methylation at the 36th lysine residue of the histone H3 (H3K36me2) and a gene expression profile that facilitates cell cycle arrest 201. This is counteracted by O-GlcNAcylation of the same serine residue to avoid cell growth arrest, even in glucose-deprived conditions 201, and is overall reminiscent of the key role of the hexosamine biosynthesis pathway, OGT and protein O-GlcNAcylation to overcome KRAS proto-oncogene, GTPase (KRAS)-induced 202 or radiation-induced senescence 203. Furthermore, O-GlcNAcylation has been shown to be important to maintain acute myeloid leukemia (AML) in an undifferentiated state 204 and has a key role in maintaining pluripotency 205, suggesting that the metabolic reprogramming that fuels the hexosamine biosynthesis pathway might be important to reactivate the genome and initiate a stem-like cell state following senescence-like reprogramming. It remains to be investigated if a nuclear shift of metabolic enzymes is involved in the epigenetic regulation of CSCs or cancer persister cells, which might represent an intriguing aspect of metabolic and epigenetic crosstalk in cancer plasticity that is yet to be explored.

Similar to the profound consequences of metabolic alteration that shape the epigenetic landscape, many metabolic pathways are regulated by epigenetic mechanisms. A quite intriguing and less explored function of KDM5 proteins is activation of genes against its well-documented gene-repressive function. This function of KDM5 in gene activation has been reported in humans and drosophila pointing to an evolutionarily conserved mechanism 206-208. Interestingly, KDM5 directly upregulates genes required for mitochondrial function in drosophila through its PHD reader motif which is distinct from its classical JmjC domain-mediated functions 207. However, this particular function of KDM5B in cancer persisters still remains ambiguous, even though KDM5B expression is shown to correlate with dependency on oxidative phosphorylation. The interdependency of epigenetic and metabolism regulation is exemplified by the direct phosphorylation of the DNA methyltransferase 1 (DNMT1), the histone acetyltransferase 1 (HAT1), and the HAT co-activator RB binding protein 7 (RBBP7) by the metabolic master regulator AMPK 209. Phosphorylation of these proteins facilitates mitochondria biogenesis by increasing the expression of peroxisome proliferator-activated receptor gamma coactivator-1α (PGC-1α), nuclear respiratory factor 1 and 2 (NRF1 and NRF2), and the mitochondrial transcription factor A (TFAM), and controls the mitochondrial membrane potential through expression of uncoupling proteins 2 and 3 (UCP2 and UCP3) 209. Furthermore, studies in *C. elegans* showed that the H3K27 demethylases JMJD-1.2 and JMJD-3.1 regulate the mitochondrial unfolded protein response to maintain mitochondrial proteostasis, which also modulates longevity in mouse models 210.

Glycolytic pathways are also subject to extensive epigenetic regulation. The EMT regulatory complex Snail-G9a-Dnmt1 was found to suppress expression of fructose-1,6-biphosphatase in basal-like breast cancer through increased histone 3 lysine 9 dimethylation (H3K9me2) and DNA methylation 211. Loss of fructose-1,6-biphosphatase, rate-limiting enzyme in gluconeogenesis, drives a shift towards increased glucose uptake and glycolysis, while inhibiting oxygen consumption and mediating the development of CSC-like characteristics 211. Furthermore, glucose uptake has been shown to be regulated by CpG island hypermethylation-linked inactivation of DERL3, a member of the protein degradation pathway, which leads to the accumulation of GLUT1 and contribution to glycolytic phenotypes in cancer cells 212. The glycolytic phenotype of cancer cell appears to be strongly mediated by epigenetic regulation as sirtuin (SIRT) 6, a H3K9 deacetylase that also controls cellular senescence 213, is a tumor suppressor that regulates aerobic glycolysis in cancer cells 214.

**Figure 3:**
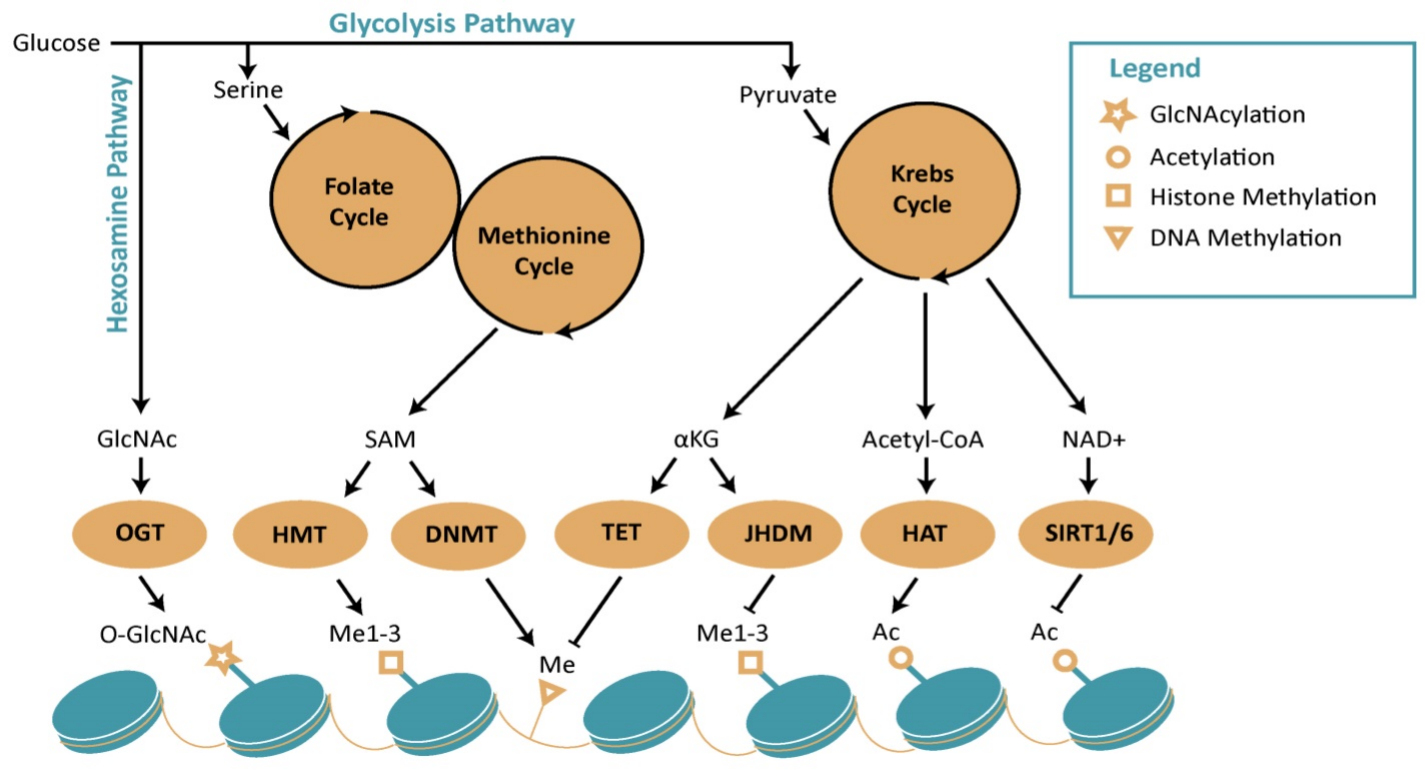
Schematic diagram representing interplay between metabolism and epigenetics. The figure represents how distinct metabolic pathways, which includes hexosamine pathway, Krebs cycle, Folate and methionine cycle, contribute to epigenetic remodeling. Abbreviations: DNMT: DNA methyl transferase; GlcNAc: N-acetylglucosamine; HAT: histone acetyltransferases; HMT: histone methyltransferases; NAD+: nicotinamide adenine dinucleotide; OGT: O-GlcNAc transferase; SAM: S-adenosylmethionine; SIRT: sirtuins; TET: ten-eleven translocation methylcytosine dioxygenase; α-KG: alpha ketoglutarate

In conclusion, the literature suggests intertwound mechanisms that connect metabolic changes to epigenetic reprogramming, which could play a central role in stress-induced phenotypic plasticity (Figure 3).

## Conclusion

Growing evidence indicates a central role for a stress-induced phenotypic plasticity in cancer progression and drug resistance. The drug-induced phenotypic plasticity leads to an initial shift of cells into a slow-cycling persister phenotype, which acts as a reservoir for the accumulation of genetic or epigenetic changes that drive the process of acquired drug resistance. This process could be critical for drug resistance to not only chemotherapy but also targeted and immune checkpoint inhibitors. Phenotypic and epigenetic plasticity is closely linked to metabolic remodeling, suggesting a close interplay between epigenetic and metabolic pathways. Furthermore, epigenetic changes are progressive rather than stochastic, suggesting a tightly regulated time-bound mechanism, which determines transient or permanent epigenetic marks contributing to the respective phenotype.

Multiple strategies have been proposed and tested to target this process of drug-induced phenotypic plasticity, which have shown promise to varying degrees. Drug holidays and intermittent treatment strategy have shown clinical efficacy 215-218. Most studies were conducted in patients who relapsed after continuous treatment of the drug. Although the patients regained partial sensitivity to the same drug after discontinuation of the drug, the precise regimen, its safety and efficacy still need further testing. Clinical trials (NCT03352947, NCT02196181) are ongoing to address these questions in melanoma patients. In addition, histone deacetylase inhibition is reported to deplete quiescent stem-like cells in multiple cancer types 219, 220, and a strategy to combine drug holidays with vorinostat, a histone deacetylase inhibitor, has shown better effects than the combination of BRAF-MEK inhibitors in a melanoma model 221. A clinical trial is ongoing in melanoma patients who showed signs of progression on BRAF-MEK inhibitor therapy (NCT02836548). A similar strategy of combining drug holidays with chemotherapeutic agent dacarbazine has also shown efficacy in melanoma pre-clinical models 104. The success of these strategies could be due to the time-dependent vulnerability induced by drug holidays during which the cells undergo phenotypic switching. Strategies targeting the metabolic reprogramming through inducing ferroptosis have also shown promise and await further testing in clinical settings 55, 98. Although many strategies that target phenotypic plasticity, epigenetics and metabolism are promising, the underlying mechanisms that determine a transient or permanent phenotype remain elusive and need further investigation. Single-cell omics analysis could be employed to dissect the heterogeneity and the transition state of stress-induced phenotypic plasticity, and further decipher transcriptional, epigenetic and metabolic crosstalks. A deeper understanding of the mechanisms that contribute to this plasticity triggered by epigenetic and metabolic changes will open a plethora of therapeutic opportunities.

## Abbreviations

ABCB5: ATP-binding cassette subfamily B member 5
ACL: ATP-citrate lyase
AFMID: arylformamidase
AhR: aryl hydrocarbon receptor
ALDH: aldehyde dehydrogenase
AML: acute myeloid leukemia
AMPK: AMP-activated protein kinase
APCDD1: APC down-regulated 1
AXL: AXL receptor tyrosine kinase
BRAF: B-Raf proto-oncogene, serine/threonine kinase
CCL2: C-C motif chemokine ligand 2
CSCs: cancer stem cells
DUSP5: dual specificity phosphatase 5
DNMT1: DNA methyl transferase 1
EGFR: epidermal growth factor receptor
EMT: epithelial-mesenchymal transition
ESC: embryonic stem cells
GPX4: Glutathione Peroxidase 4
GlcNAc: N-acetylglucosamine
HAT1: histone acetyltransferases 1
HMT: histone methyltransferases
hPSC: human pluripotent stem cells
H3K4me3: histone 3 lysine 4 trimethylation
H3K9me3: histone 3 lysine 9 trimethylation
H3K9me2: histone 3 lysine 9 dimethylation
H3K27: histone 3 lysine 27
H3K27me3: histone 3 lysine 27 trimethylation
H3K36me3: histone 3 lysine 36 trimethylation
H3K36me2: histone 3 lysine 36 dimethylation
IDO: indoleamine-2,3-dioxygenase
IFN-γ: interferon-γ
IPRES: innate anti-PD-1 resistance signature
iPSC: induced pluripotent stem cell
JARID1: Jumonji AT-rich interactive domain 1
JmjC: Jumonji C
KDM5A: lysine-specific demethylase 5A
KDM5B: lysine-specific demethylase 5B
KRAS: KRAS Proto-Oncogene, GTPase
MAPK: mitogen-activated protein kinase
MHC: major histocompatibility complex
NAD+: nicotinamide adenine dinucleotide
NAMPT: nicotinamide phosphoribosyltransferase
NGFR: nerve growth factor receptor
NRAS: neuroblastoma RAS Viral (V-Ras) Oncogene Homolog
NRF1: nuclear respiratory factor 1
NRF2: nuclear respiratory factor 2
NRG1: neuregulin 1
NSG: NOD scid gamma mouse
OGT: O-GlcNAc transferase
OXPHOS: oxidative phosphorylation
PD1: programmed cell death protein 1
PDGFRB: platelet-derived growth factor receptor beta
PDH: pyruvate dehydrogenase
PDK1: pyruvate dehydrogenase kinase 1
PDP2: pyruvate dehydrogenase phosphatase 2
PGC-1α: peroxisome proliferator-activated receptor gamma coactivator-1α
PHGDH: phosphoglycerate dehydrogenase
PUFA: polyunsaturated fatty acids
RBBP7: RB binding protein 7
SAHF: senescent associated heterochromatin foci
SAM: S-adenosylmethionine
SCID: severe combined immunodeficiency
SETDB1: SET domain bifurcated histone lysine methyltransferase 1
SETDB2: SET domain bifurcated histone lysine methyltransferase 2
SGOCP: serine, glycine, one-carbon pathway
SIPS: stress-induced premature senescence
SIRT: sirtuin
TDO: tryptophan-2,3-dioxygenase
TET: ten-eleven translocation methylcytosine dioxygenase
TFAM: mitochondrial transcription factor A
TIC: tumor initiating lung cancer stem cells
TRC: tumor repopulating cells
VEGF: vascular endothelial growth factor
ZGA: zygotic genome activation
α-KG: alpha ketoglutarate
